# Domain-specific physical activity and risk of suicidal ideation in adults: a population-based study

**DOI:** 10.1186/s12889-025-23815-9

**Published:** 2025-07-29

**Authors:** Yun Zhang, Tong Xu, Zicheng Cheng, Fangwang Fu, Zhenxiang Zhan, Lili Ma, Meiqi Zhao

**Affiliations:** 1 Department of Neurology, Affiliated Jinhua Hospital, Zhejiang University School of Medicine, Jinhua, China; 2 Department of Neurology, The Second Affiliated Hospital and Yuying Children’s Hospital of Wenzhou Medical University, Wenzhou, Zhejiang Province 325600 China

**Keywords:** Domain-specific physical activity, Leisure-time physical activity, Intensity, Mental health, National health and nutrition examination survey, Suicidal ideation

## Abstract

**Background:**

It remains unclear whether all physical activity (PA) domains (occupational PA [OPA], transportation PA [TPA], and leisure-time PA [LTPA]) have equivalent beneficial effects. We aimed to investigate the associations of OPA, TPA, and LTPA with suicidal ideation in adults.

**Methods:**

We included and analyzed 25,497 participants (aged ≥ 18 years) from the National Health and Nutrition Examination Survey (NHANES) 2007–2018. The domain-specific PA was assessed by a self-report questionnaire and categorized based on the PA guidelines. Suicidal ideation was measured by item nine of the nine-item Patient Health Questionnaire.

**Results:**

Participants achieving PA guidelines (≥ 150 min/week) had a 21% (odds ratio [OR] 0.79, 95% confidence interval [CI] 0.63–0.98) and 32% (OR 0.68, 95% CI 0.53–0.89) reduced likelihood of suicidal ideation depending on total PA and LTPA, respectively, while OPA or TPA did not correlated with suicidal ideation. The mediation analysis revealed that 74.1% of this association was mediated by the severity of depressive symptoms. In subgroup analyses, LTPA was associated with suicidal ideation in participants who were aged ≥ 60 years, those with BMI < 30 kg/m^2^, sedentary time < 360 min/day, smoking, hypertension, depression and proportion of vigorous exercise ≥ 0.5. These connections indicate that LTPA could be especially beneficial for older individuals and those with specific lifestyle or health risks. LTPA at levels of 150–299 and ≥ 300 min/week was associated with 31% (OR 0.69, 95% CI 0.49–0.97) and 30% (OR 0.70, 95% CI 0.52–0.94) lower odds of suicidal ideation, respectively. Stratified by exercise intensity, vigorous-intensity LTPA, but not moderate-intensity LTPA, was negatively associated with the risk of suicidal ideation (OR 0.61, 95% CI 0.44–0.86).

**Conclusions:**

When the amount achieving the PA guidelines, LTPA, but not OPA or TPA, was associated with a lower risk of suicidal ideation through the mediation of depressive symptoms. These findings indicate that engaging in LTPA, especially vigorous intensity LTPA, may provide substantial benefits for mental health.

**Supplementary Information:**

The online version contains supplementary material available at 10.1186/s12889-025-23815-9.

## Introduction

Globally, suicide is a public health issue that results in over 700,000 fatalities annually [1]. The World Health Organization (WHO) has listed a one-third reduction in the rate of suicide by 2030 as an action plan [2]. Suicidal ideation appears before suicidal attempts, which eventually leads to suicide, according to ideation-to-action theories of suicide [3]. Accordingly, targeted intervention strategies are essential to prevent or treat suicidal ideation. The reported lifestyle factors related to suicidal ideation include physical activity (PA), diet, nicotine exposure, and sleep health [4–6]. Among these modifiable lifestyles, PA has been shown to potentially reduce suicidal behaviors [7].

PA represents a complex behavior consisting of various domains: occupational PA (OPA), transportation PA (TPA), and leisure-time PA (LTPA) [8]. Different PA domains may have different impacts on mental health, including depression, anxiety, and stress [9]. For example, two cross-sectional studies in East Asia reported that LTPA and TPA are inversely associated with depressive symptoms, whereas OPA may increase the risk of depression, suggesting the mental health benefits of physical activity depend critically on its domain-specific context [10, 11]. While existing studies have established the association between total PA and suicidal ideation in youth populations [12–16], the domain-specific effects across diverse groups, such as age and sex, remain underexplored. A recent systematic review by Vancampfort et al. highlighted that 89.7% of studies on PA and suicidal ideation focused on adolescents or adults, with only 10.3% including older adults over 60 years [17]. Notably, a cross-sectional study examined the association between PA and suicidal ideation and attempts among older adults in five developing countries, finding that insufficient PA, especially LTPA, was significantly associated with a higher risk of suicidal ideation and attempts, with variations observed across genders and countries [18]. This suggests domain-specific impacts may vary with gender and ethnic context. These findings collectively underscore critical gaps in existing evidence regarding differential effects of PA of suicidal ideation depending on specific PA domains and various population.

As there are limited studies distinguishing the beneficial effects of OPA, TPA, and LTPA on suicidal ideation, our study aims to address this gap in knowledge by exploring the associations between various PA domains and the risk of suicidal ideation in adults across a wide age spectrum. Further analysis was conducted to determine if the association between domain-specific PA and suicidal ideation varied among distinct groups. In addition, we evaluated the dose–response relationship between distinct PA domains and suicidal ideation.

## Methods

### Study population

The National Health and Nutrition Examination Survey (NHANES), designed and performed by the National Center for Health Statistics (NCHS), aims to study the health and nutritional status of United States (US) individuals. As a continuous program, this complex, multistage probability sampling survey selects a nationally representative sample of nearly 5000 individuals yearly. During the interviews, participants are asked a series of questions about their sociodemographic background, dietary habits, and health. They also undergo thorough medical, dental, laboratory, and physiological examinations. The NCHS Ethics Review Board authorized the NHANES protocols, with all participants signing informed consent.

**Fig. 1:**
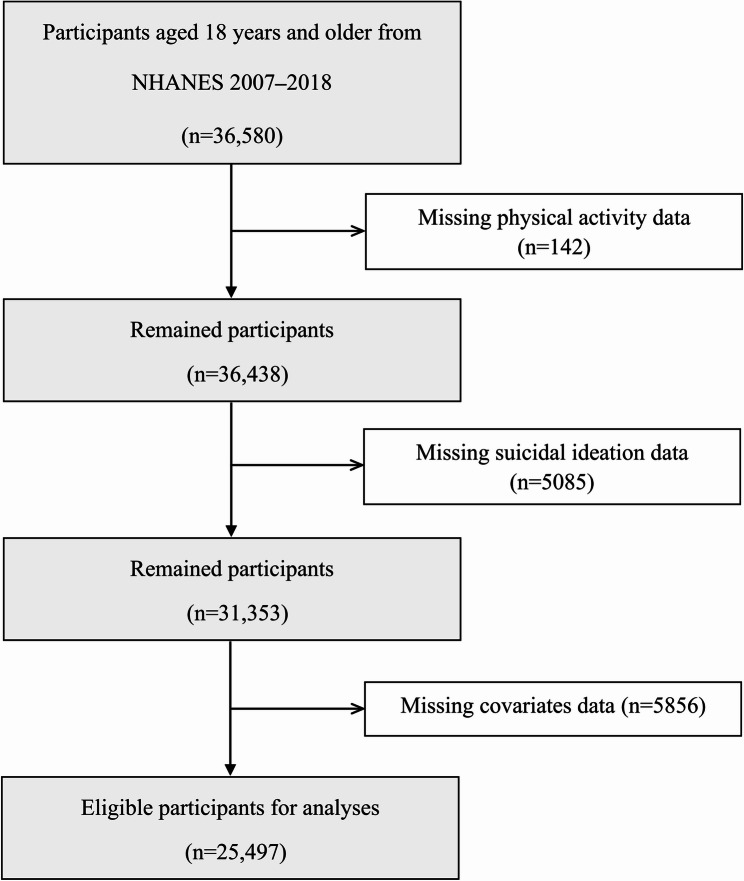
Flow diagram of the included survey participants

This is a cross-sectional study because of using NHANES data. The original inclusion for this study encompassed all participants from the NHANES 2007–2018 cycles aged 18 years or older, resulting in 36,580 individuals. After excluding participants without complete PA, suicidal ideation, and related covariates data, our study recruited 25,497 individuals to perform complete-case analysis (Fig. 1). The datasets used in this study are publicly available in the National Health and Nutrition Examination Survey repository at https://wwwn.cdc.gov/nchs/nhanes/default.aspx.

### Physical activity

Trained interviewers collected the data on PA through the Global Physical Activity Questionnaire evaluating three PA domains: OPA, TPA, and LTPA [19]. The questionnaire covers PA frequency (days/week), duration (minutes/day), and intensity (moderate or vigorous) in various domains within a typical week. The frequency and duration were multiplied to calculate the minutes of PA for each domain and intensity of PA. According to World Health Organization (WHO) guidelines recommending at least 150–300 min of moderate-intensity aerobic PA, 75–150 min of vigorous-intensity aerobic PA, or an equivalent combination of both throughout the week, we allocated the participants into PA achieved and unachieved groups. The intensity of OPA and LTPA includes both moderate and vigorous, while TPA is only moderate in intensity. Therefore, the compliance status for PA, OPA, and LTPA is categorized into three scenarios: ​​overall compliance (≥ 150 min/week)​​, ​​moderate-intensity compliance​​ (≥ 150 min/week), and ​​vigorous-intensity compliance (≥ 75 min/week)​​. To calculate overall minutes for OPA or LTPA, the minutes of the vigorous intensity were doubled and added to the minutes of the moderate intensity. The sum of the OPA, TPA, and LTPA results in the total PA. According to earlier reports, the summed minutes of PA were assigned into (1) 0, (2) 1–149, (3) 150–299, and (4) ≥ 300 min/week groups to ascertain the dose-response relationship between related PA domains and suicidal ideation [20, 21]. Additionally, we also summed the minutes of moderate- and vigorous-intensity PA directly as a supplementary analysis for dose-response relationship.

### Suicidal ideation

The nine-item Patient Health Questionnaire (PHQ-9) is widely used to estimate respondents’ psychological well-being during the last two weeks [22]. Suicidal ideation was evaluated by analyzing the answer to item nine of the PHQ-9, which asked: “Over the last two weeks, how often have you felt that you would be better off dead, or hurting yourself in some way?“. Participants answering never were classified into the non**-**suicidal ideation group; otherwise, into the suicidal ideation group. Removing item nine, the first eight items (PHQ-8) were employed to compute a depression score [23]. A score ≥ 10 was considered as having depressive symptoms.

### Covariates

Covariates of interest including sociodemographic, lifestyle, and health status were deemed potential confounders. Sociodemographic variables comprised age, sex (female, male), race (Mexican American, other Hispanic, non-Hispanic White, non-Hispanic Black, other race), education level (less than high school, high school, more than high school), marital status (married/living with partner, widowed/divorced/separated, never married), and the family income-to-poverty ratio. Lifestyle variables included smoking, drinking, and sedentary time. Smoking history was characterized by consuming a minimum of 100 cigarettes throughout one’s lifetime. Drinking history was the practice of consuming a minimum of 12 alcoholic drinks throughout one year. Health status includes body mass index (BMI), waist circumference, hypertension (HTN), diabetes mellitus (DM), hyperlipidemia, coronary heart disease, stroke, cancer, and depression. The diagnostic criteria for the diseases mentioned above were consistent with our previous research [24].

### Statistical analysis

The unweighted frequency and weighted percentage were utilized to represent categorical variables, while using the weighted median and interquartile range (IQR) to depict continuous variables. The analysis of intergroup differences of continuous and categorical variables was conducted by the Wilcoxon rank–sum test and the chi-squared test with Rao and Scott’s second-order correction, respectively. Logistic regression models were employed for evaluating the association between PA and risk of suicidal ideation. No adjustments were made to any covariates in the Unadjusted Model. Age, sex, and race were adjusted in Model 1. Model 2 was additionally adjusted to account for other covariates having *p* < 0.1 in the univariate analysis. A variance inflation factor (VIF) greater than 10 suggests multicollinearity among the independent variables included in the model. Akaike Information Criterion (AIC) was used to compare model fit and complexity across different models. The discrimination of model was assessed using the area under the receiver operating characteristic (AUROC) curve to reflect the ability to distinguish between those with and without suicidal ideation. The calibration of model was evaluated using the Brier score, reflecting the consistency between the model’s predicted probability and observed results in the data. Internal validation was performed using the bootstrap method, which involved 1000 resampling iterations to generate 1000 training sets, with the original training set serving as the validation set to assess the model’s optimism and statistical performance. Mediation analysis was employed to estimate the possible mediating implications of depressive severity (PHQ-8 score) on the association between LTPA and suicidal ideation [25]. First, we used a linear regression model to examine the association between the independent variable (LTPA) and the mediator variable (PHQ-8 score), adjusting for all relevant covariates. Then, we fitted a logistic regression model to analyze the effects of both the independent variable (LTPA) and mediator (PHQ-8 score) on the outcome variable (suicidal ideation), while controlling for the same set of covariates. Finally, we conducted mediation analysis to estimate the direct, indirect, and total effects using the counterfactual framework with bootstrap confidence intervals. Stratified analyses were performed to investigate correlations in various subgroups, including age (< 60/≥ 60 years), sex (female/male), BMI (< 30/≥ 30 kg/m^2^), smoking (no/yes), sedentary time (< 360/≥ 360 min/day), HTN (no/yes), DM (no/yes), depression (no/yes), and proportion of vigorous exercise (< 0.5/≥ 0.5). As a sensitivity analysis, we performed an ordinal logistic regression analysis using the original score of PHQ-9 item nine as the outcome to examine the association between PA and risk of suicidal ideation. The statistical analyses were carried out employing R version 4.2.1 (R Foundation for Statistical Computing, Vienna, Austria), setting a two-tailed *p* < 0.05 as statistical significance. Sample weights were utilized to illustrate the complex sampling design of the NHANES [26].

## Results

Table 1 shows the detailed information of the study population according to whether they had suicidal ideation. Of these participants, the median age was 47.0 (IQR: 33.0–60.0) years and females accounted for 50.9%. Overall, 949 (3.7%) participants were identified as having suicidal ideation in this population. The median minutes of total PA, OPA, TPA, and LTPA were 360.0 (IQR: 60.0–1170.0), 0.0 (IQR: 0.0–600.0), 0.0 (IQR: 0.0–0.0), 60.0 (IQR: 0.0–300.0), respectively. According to the WHO PA guidelines, 15,629 (66.1%) participants were able to meet the recommended total PA amount (≥ 150 min/week). In addition, 8952 (38.6%), 3531 (12.7%), and 8641 (38.5%) participants fulfilled the recommended OPA, TPA, and LTPA amounts, respectively. The compliance rates of moderate-intensity PA, OPA, and LTPA and vigorous-intensity PA, OPA, and LTPA were 55.2%, 33.5%, 22.3%, 36.8%, 19.5%, and 22.6%, respectively.

**Table 1:** Participants’ characteristics stratified by the presence of suicidal ideation

	Overall (*N* = 25,497)	Non-suicidal ideation (*N* = 24,548)	Suicidal ideation (*N* = 949)	*P*-value
Age	47.0 (33.0–60.0)	47.0 (33.0–60.0)	47.0 (34.0–59.0)	0.97
Sex				0.14
Female	12,857 (50.9%)	12,346 (50.8%)	511 (54.1%)	
Male	12,640 (49.1%)	12,202 (49.2%)	438 (45.9%)	
Race				< 0.001
Mexican American	3715 (8.1%)	3556 (8.1%)	159 (9.0%)	
Other Hispanic	2548 (5.4%)	2399 (5.3%)	149 (9.6%)	
Non-Hispanic White	11,057 (68.6%)	10,670 (68.8%)	387 (63.0%)	
Non-Hispanic Black	5350 (10.6%)	5186 (10.6%)	164 (10.5%)	
Other Race	2827 (7.3%)	2737 (7.3%)	90 (7.9%)	
Education level				< 0.001
Less than high school	5742 (14.5%)	5395 (14.1%)	347 (25.2%)	
High school	5865 (22.9%)	5652 (22.9%)	213 (24.0%)	
More than high school	13,890 (62.6%)	13,501 (63.0%)	389 (50.8%)	
Marital status				< 0.001
Married/living with partner	15,242 (63.8%)	14,822 (64.4%)	420 (47.7%)	
Widowed/divorced/separated	5597 (18.1%)	5285 (17.8%)	312 (29.0%)	
Never married	4658 (18.1%)	4441 (17.9%)	217 (23.3%)	
Family income-to-poverty ratio	3.0 (1.5–5.0)	3.1 (1.5–5.0)	1.7 (0.9–3.2)	< 0.001
Body mass index (kg/m²)	28.0 (24.3–32.6)	28.0 (24.3–32.6)	29.1 (24.3–33.5)	0.017
Waist circumference (cm)	98.2 (87.5–109.6)	98.1 (87.5–109.5)	101.5 (90.3–112.0)	< 0.001
Smoking	11,576 (45.0%)	11,022 (44.6%)	554 (57.7%)	< 0.001
Drinking	17,657 (74.3%)	17,001 (74.4%)	656 (72.3%)	0.31
Hypertension	10,632 (36.7%)	10,186 (36.5%)	446 (42.0%)	0.022
Diabetes mellitus	4329 (12.5%)	4117 (12.4%)	212 (16.6%)	< 0.001
Hyperlipidemia	14,641 (57.2%)	14,046 (57.0%)	595 (62.8%)	0.003
Coronary heart disease	1012 (3.3%)	947 (3.3%)	65 (5.6%)	0.002
Stroke	904 (2.6%)	833 (2.5%)	71 (6.2%)	< 0.001
Cancer	2479 (10.3%)	2365 (10.3%)	114 (12.6%)	0.067
Depression	4143 (17.3%)	3512 (15.7%)	631 (66.4%)	< 0.001
PA: achieved	15,629 (66.1%)	15,147 (66.5%)	482 (54.4%)	< 0.001
Moderate-intensity PA: achieved	13,171 (55.2%)	12,761 (55.5%)	410 (46.1%)	< 0.001
Vigorous-intensity PA: achieved	8241 (36.8%)	8003 (37.2%)	238 (26.8%)	< 0.001
OPA: achieved	8952 (38.6%)	8637 (38.7%)	315 (38.1%)	0.82
Moderate-intensity OPA: achieved	7623 (33.5%)	7371 (33.6%)	252 (30.7%)	0.18
Vigorous-intensity OPA: achieved	4551 (19.5%)	4382 (19.5%)	169 (19.7%)	0.90
TPA: achieved	3531 (12.7%)	3396 (12.7%)	135 (12.8%)	0.99
LTPA: achieved	8641 (38.5%)	8447 (39.1%)	194 (22.6%)	< 0.001
Moderate-intensity LTPA: achieved	4807 (22.3%)	4711 (22.7%)	96 (10.5%)	< 0.001
Vigorous-intensity LTPA: achieved	5204 (22.6%)	5080 (22.9%)	124 (15.0%)	< 0.001
Sedentary time (minutes/day)	360.0 (240.0–480.0)	360.0 (240.0–480.0)	360.0 (240.0–540.0)	0.32

*LTPA* leisure-time physical activity, *OPA* occupational physical activity, *PA* physical activity, *TPA* transportation physical activity

Table 2 presents the results of logistic regression analyses between various PA domains meeting PA guidelines and the risk of suicidal ideation. We found that total PA (odds ratio [OR] 0.60, 95% confidence interval [CI] 0.50–0.73) and LTPA (OR 0.46, 95% CI 0.36–0.58) achieving PA guidelines exhibited an inverse association with the risk of suicidal ideation in the Unadjusted Model. In Model 1, the associations of total PA (OR 0.60, 95% CI 0.49–0.74) and LTPA (OR 0.46, 95% CI 0.36–0.58) with suicidal ideation were still significant. Model 2 revealed that those meeting PA guidelines for total PA displayed a 21% reduced likelihood of exhibiting suicidal ideation (OR 0.79, 95% CI 0.63–0.98), and those achieving the PA guidelines for LTPA possessed a 32% reduction in the possibility of displaying suicidal ideation (OR 0.68, 95% CI 0.53–0.89). Nonetheless, both OPA and TPA were unrelated to suicidal ideation in any model. The smallest AIC value of Model 3 containing LTPA indicated that it was the best fitted model. An AUROC of 0.823 and a brier score of 0.032 of this model indicated strong discrimination and calibration. Bootstrapped validation results regarding AUROC, 0.820 (95% CI 0.815–0.823), and brier score, 0.033 (95% CI 0.032–0.033), indicated that the optimism of this model was negligible. Further analysis based on exercise intensity revealed that vigorous-intensity LTPA was the only significant factor negatively associated with the risk of suicidal ideation (OR 0.61, 95% CI 0.44–0.86) (Table 3).

**Table 2:** Logistic regression analysis to identify the association between domain-specific PA and suicidal ideation

	Event/*N* (%) *	Unadjusted Model			Model 1			Model 2		
		OR (95% CI)	*P*–value	AIC	OR (95% CI)	*P*–value	AIC	OR (95% CI)	*P*–value	AIC
Total PA: achieved										
No	467/9868 (4.4%)	1 (Ref)			1 (Ref)			1 (Ref)		
Yes	482/15,629 (2.7%)	0.60 (0.50–0.73)	< 0.001	7291.7	0.60 (0.49–0.74)	< 0.001	7279.0	0.79 (0.63–0.98)	0.032	6091.9
Occupational PA: achieved										
No	634/16,545 (3.3%)	1 (Ref)			1 (Ref)			1 (Ref)		
Yes	315/8952 (3.2%)	0.98 (0.80–1.19)	0.82	7341.8	1.01 (0.82–1.25)	0.91	7325.2	1.04 (0.84–1.29)	0.69	6100.4
Transportation PA: achieved										
No	814/21,966 (3.3%)	1 (Ref)			1 (Ref)			1 (Ref)		
Yes	135/3531 (3.3%)	1.00 (0.76–1.33)	0.99	7341.8	0.99 (0.75–1.32)	0.96	7324.8	0.93 (0.68–1.26)	0.62	6100.1
Leisure-time PA: achieved										
No	755/16,856 (4.1%)	1 (Ref)			1 (Ref)			1 (Ref)		
Yes	194/8641 (1.9%)	0.46 (0.36–0.58)	< 0.001	7242.9	0.46 (0.36–0.58)	< 0.001	7229.9	0.68 (0.53–0.89)	0.005	6083.5

Model 1: adjusted for age, sex, and race

Model 2: adjusted for age, sex, race, education level, marital status, family income-to-poverty ratio, body mass index, waist circumference, smoking, hypertension, diabetes mellitus, hyperlipidemia, coronary heart disease, stroke, cancer, and depression

*CI* confidence interval, *OR* odds ratio, *PA* physical activity

*The method to address missing data was using complete-case analysis

**Table 3:** Logistic regression analysis to identify the association between different intensity of domain-specific PA and suicidal ideation

	Event/*N* (%)	OR (95% CI)	*P*–value
Moderate-intensity PA: achieved	410/13,171 (2.7%)	0.84 (0.68 − 1.03)	0.10
Vigorous-intensity PA: achieved	238/8241 (2.4%)	0.86 (0.67 − 1.09)	0.21
Moderate-intensity OPA: achieved	252/7623 (3.0%)	0.91 (0.71 − 1.18)	0.48
Vigorous-intensity OPA: achieved	169/4551 (3.3%)	1.11 (0.83 − 1.51)	0.47
Moderate-intensity LTPA: achieved	96/4807 (1.5%)	0.85 (0.65 − 1.11)	0.22
Vigorous-intensity LTPA: achieved	124/5204 (2.2%)	0.61 (0.44 − 0.86)	0.005

Adjusted for the same covariates in Model 2

*LTPA* leisure-time physical activity, *OPA* occupational physical activity, *PA* physical activity, *TPA* transportation physical activity

**Fig. 2:**
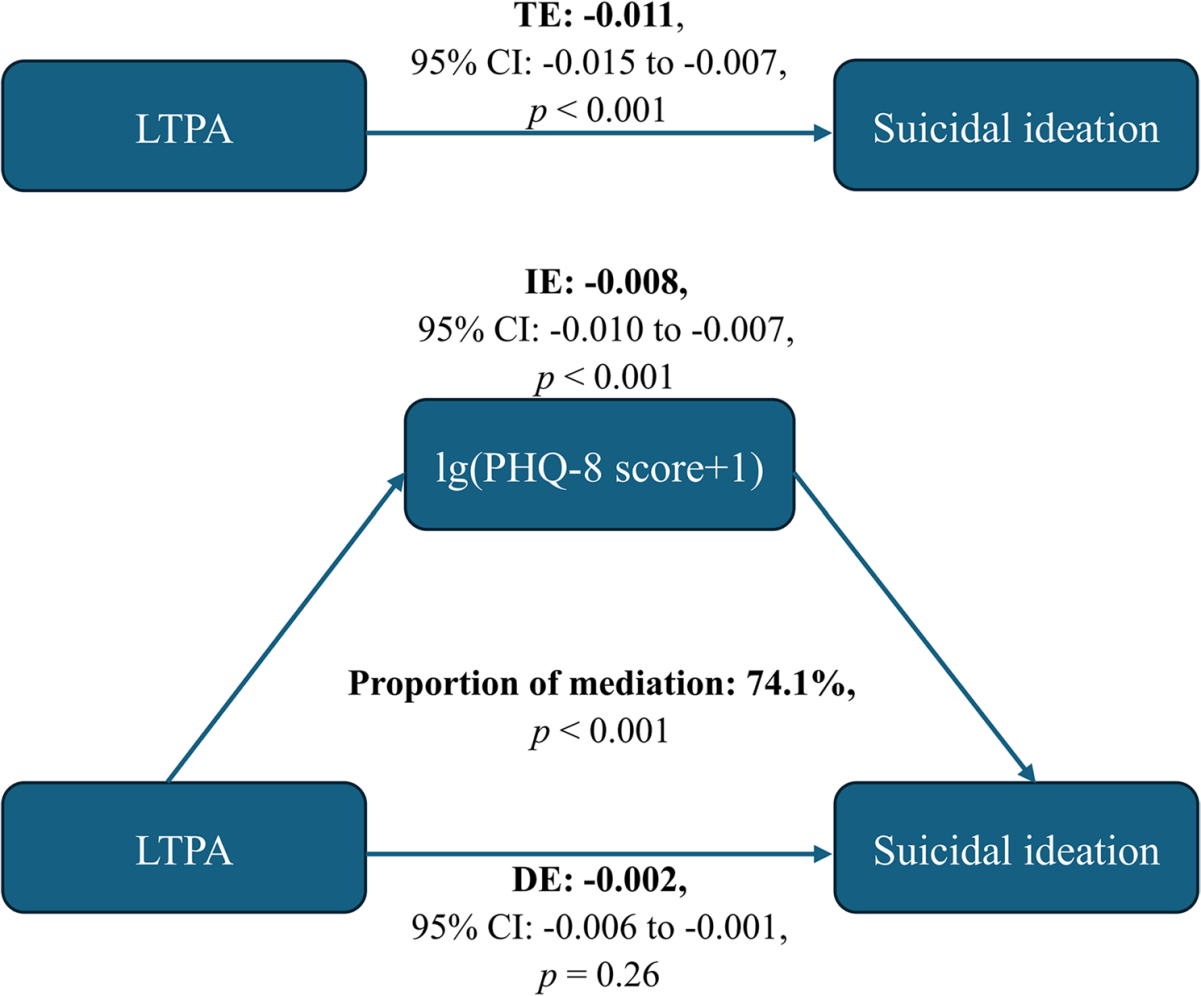
Mediating effects of depressive symptoms on the association between LTPA and the risk of suicidal ideation. CI, confidence interval; DE, direct effect; IE, indirect effect; LTPA, leisure-time physical activity; PHQ, Patient Health Questionnaire; TE, total effect

As shown in Fig. 2, the total effect represented the implication of LTPA on suicidal ideation (*p* < 0.001); the direct effect represented the implication of LTPA on suicidal ideation, not mediated by PHQ-8 score (*p* = 0.26); the indirect effect represented the implication of LTPA on suicidal ideation, mediated by PHQ-8 score (*p* < 0.001). The proportion of PHQ-8 score mediating the effect of LTPA on suicidal ideation was 74.1%. The additional analysis on the association between PA and depression yielded similar results, showing that reduced depression risk was associated with LTPA rather than OPA or TPA (Supplementary Table 1).

**Fig. 3:**
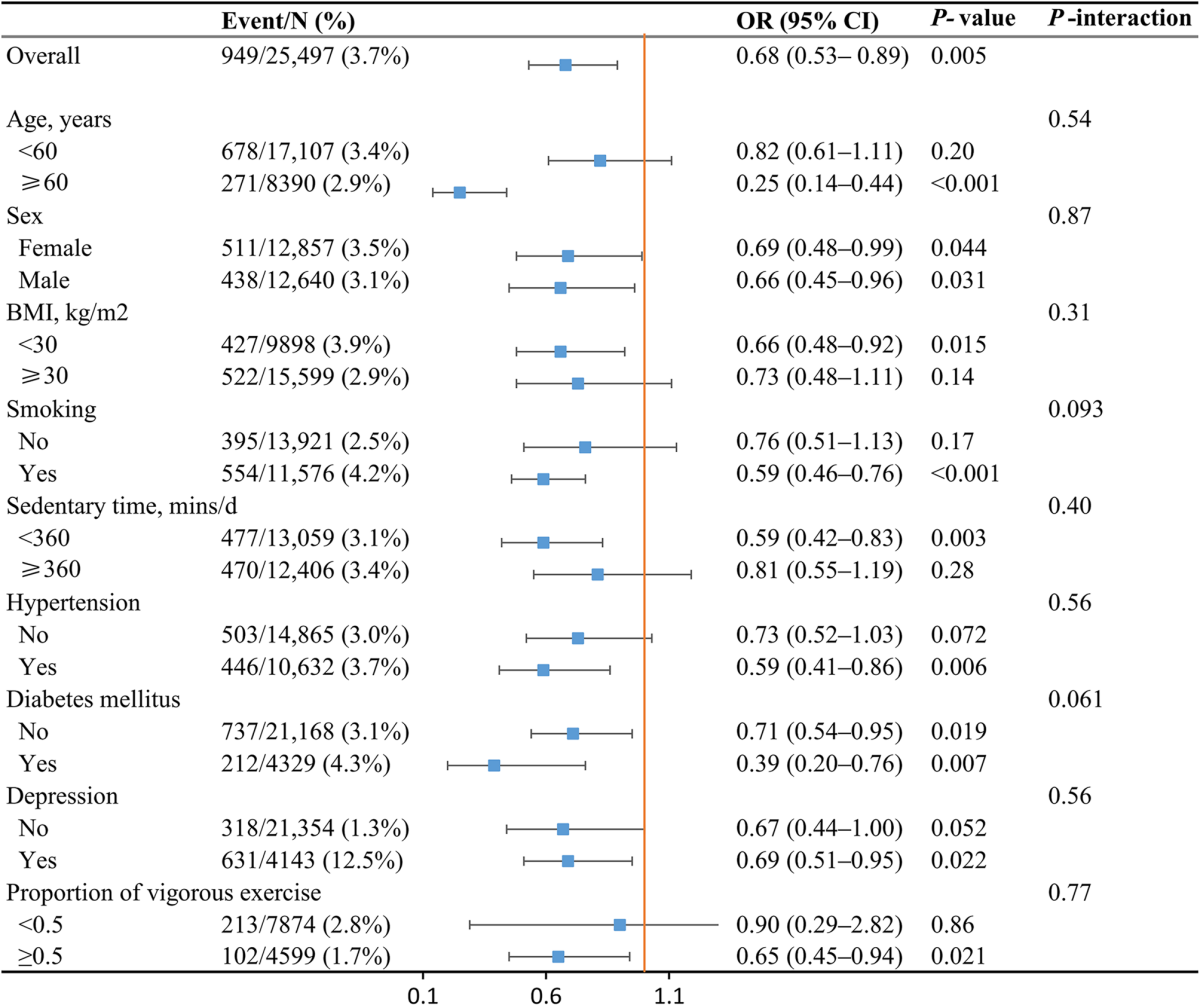
Association between LTPA meeting PA guidelines and risk of suicidal ideation in subgroups. CI, confidence interval; OR, odds ratio; PA, physical activity; LTPA, leisure-time physical activity. All ORs were adjusted for the same covariates in Model 2

Figure 3 shows the stratified analyses for the association between LTPA and suicidal ideation in multivariable Model 2. Except for sex and DM, the significant association between LTPA and suicidal ideation existed in participants with aged ≥ 60 years (OR 0.25, 95% CI 0.14–0.44), BMI < 30 kg/m^2^ (OR 0.66, 95% CI 0.48–0.92), sedentary time < 360 min/day (OR 0.59, 95% CI 0.42–0.83), smoking (OR 0.59, 95% CI 0.46–0.76), hypertension (OR 0.59, 95% CI 0.41–0.86), depression (OR 0.69, 95% CI 0.51–0.95), and proportion of vigorous exercise ≥ 0.5 (OR 0.65, 95% CI 0.45–0.94). In those without severe depression, that is PHQ-8 score ranging 0–14, LTPA was still significantly associated with suicidal ideation (OR 0.60, 95% CI 0.45–0.80).

Among participants with suicidal ideation, 665 (71.4%) reported it for several days, 146 (15.9%) for more than half the days, and 138 (12.7%) nearly every day in the past two weeks.​ Ordinal logistic regression model showed that LTPA, rather than OPA or TPA, was associated with lower order of severity of suicidal ideation (OR 0.69, 95% CI 0.53–0.89) (Supplementary Table 2).

**Fig. 4:**
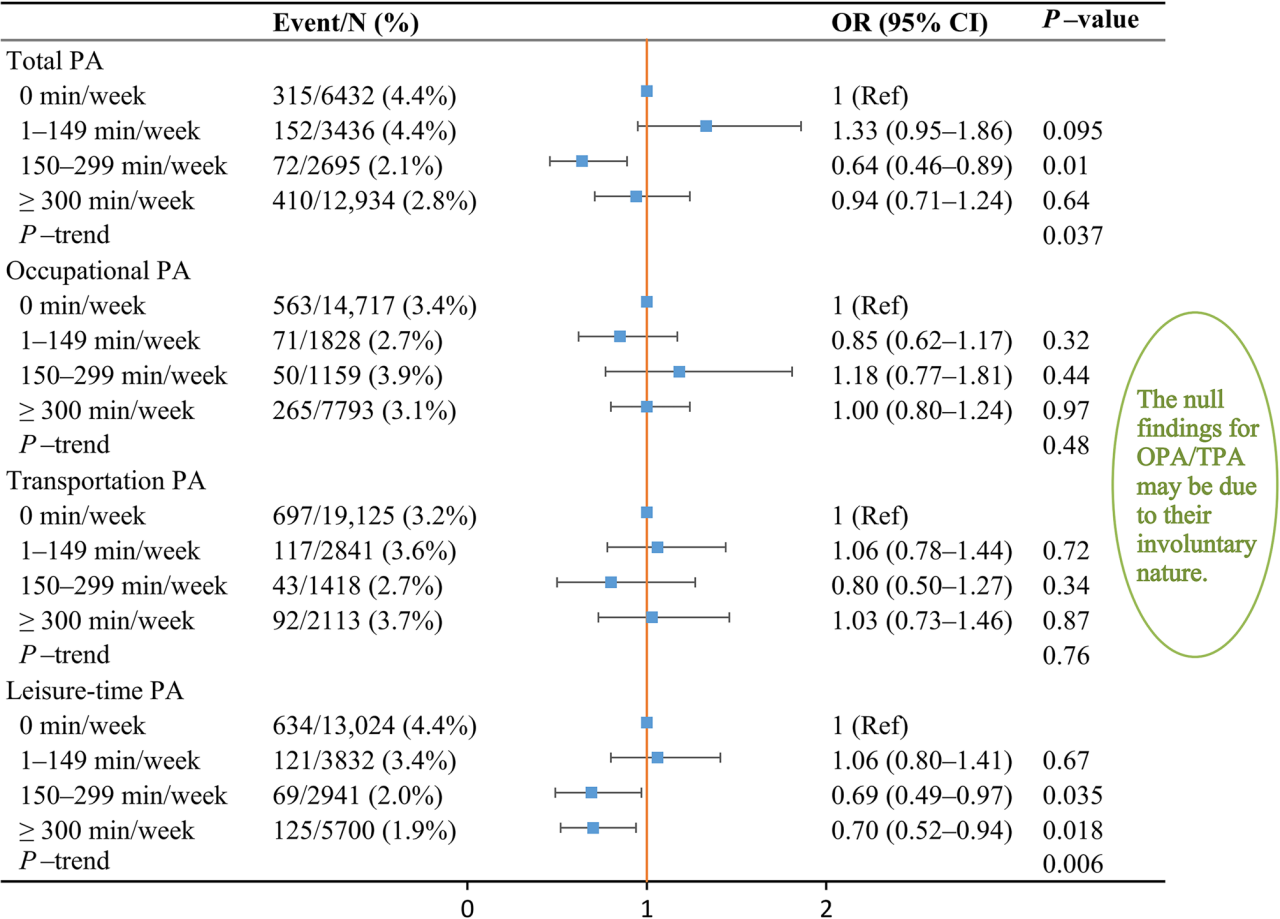
Multivariable OR for suicidal ideation based on the amount of PA with applying the 2× weighting for vigorous-intensity PA. CI, confidence interval; OR, odds ratio; PA, physical activity. All ORs were adjusted for the same covariates in Model 2

In addition, PA amount was categorized into four groups (0, 1–149, 150–299, and ≥ 300 min/week) to ascertain the probable dose-response relationships between various PA domains and suicidal ideation, in order to evaluate further PA benefits beyond or below the PA guidelines (Fig. 4). Compared with participants without LTPA (0 min/week), the adjusted ORs of suicidal ideation for participants performing < 1 time (1–149 min/week), 1–2 times (150–299 min/week), or > two times (≥ 300 min/week) of the advocated amount of PA guidelines were 1.06 (95% CI 0.80–1.41), 0.69 (95% CI 0.49–0.97), and 0.70 (95% CI 0.52–0.94), respectively. This implies that LTPA was related to a lower risk of suicidal ideation when the amount of LTPA achieved PA guidelines. However, no significant associations of OPA and TPA with suicidal ideation were recognized (*p* > 0.05). Supplementary dose-response analysis yielded similar results, and the protective effect of LTPA on the risk of suicidal ideation appeared at ≥ 300 min/week (Supplementary Fig. 1).

## Discussion

This US nationally representative cross-sectional survey elucidated that LTPA fulfilling the WHO PA guidelines was independently related to reduced risk and severity of suicidal ideation. Mediation analysis indicated that this association was not direct and mediated by the severity of depression. Dose–response analysis suggested that LTPA levels below the recommendations was not associated with lower the risk of suicidal ideation, while LTPA levels above the recommendations failed to further reduce the risk of suicidal ideation. Further stratification revealed that ​​vigorous-intensity LTPA​​ had a stronger association with reduced risk of suicidal ideation compared to ​​moderate-intensity LTPA. The results showed no significant associations of OPA and TPA with suicidal ideation.

Our study has several strengths. First, the reports on the beneficial effect of total PA on suicidal ideation usually focused on adolescents [12, 14, 16]. Our findings extended this beneficial effect to the general adult population. Consistent results were observed in the general population of South Korea, where the beneficial effect was only observed in the moderate total PA group compared to the low total PA group, and not in the high total PA group [13]. A reasonable explanation is that the high total PA group includes high levels of OPA and LPA, which weakens the link between PA and suicidal ideation. Second, the differences in the health influences of distinct PA domains were elaborated in our study. Previous studies have seldom distinguished the effects of different PA domains. A cross-sectional survey covering elderly participants from five countries showed that not engaging in LTPA, OPA, or TPA was more likely to have suicidal ideation, which was influenced by gender and country [18]. However, the sample size of some countries has insufficient statistical power, and the simple pooled analysis ignores the population heterogeneity between different countries. Third, subgroup and dose–response analyses were performed to offer comprehensive data on the health effects of PA. The results have filled the evidence gaps in existing research and followed the previously proposed recommendations for future research [8].

LTPA encompasses a wide range of activities, such as sports, fitness, or leisure activities, that individuals engage in during their free time, according to their personal interests and requirements [27]. The beneficial effect of LTPA on psychological health may involve the downregulation of pro-inflammatory cytokine expression and the increase of neurotransmitters, such as serotonin and endorphins [28]. Ghose et al. analyzed cross-sectional data of 2861 participants aged 50 years or above and found that lacking moderate intensity LTPA had an increased risk of suicidal ideation, especially among women [18]. A recent cross-sectional study of 4080 military personnel reported that participating in ≥ 150 min/week moderate-intensity LTPA is inversely associated with suicide ideation, regardless of age, sex, and unhealthy lifestyles [29]. However, none of these studies accounted for ​​vigorous-intensity LTPA​​. Logistic regression model incorporating both ​​moderate- and vigorous-intensity LTPA, along with subgroup analysis based on the proportion of vigorous exercise in our study, demonstrated that ​​vigorous-intensity LTPA​​ was more strongly associated with suicidal ideation than ​​moderate-intensity LTPA. These findings provide support for the notion that engaging in LTPA may be an effective strategy for preventing suicidal ideation.

Depression represents a major risk factor of suicidal ideation, and the prevalence of suicidal ideation is significantly higher in the depressed population [30]. A recent survey among South Korean adolescents revealed that depression mediated the negative association between LTPA and suicidal ideation [31]. Our study, through mediation analysis, further elucidated that the negative association between LTPA and suicidal ideation was indirect and fully mediated by the severity of depression in general adults. Although the effects of OPA and TPA on depression are inconsistent among different studies, the improvement of depression by LTPA has been consistently concluded in previous studies [10, 11, 21, 32]. In our study, the domain-specific PA associated with both depression and suicidal ideation risk was exclusively LTPA, rather than OPA or TPA. This consistency supports the mediating role of depression in the association between LTPA and suicidal ideation.

Our study identified several factors influencing the relationship between LTPA and suicidal ideation, such as older age, lower BMI and sedentary time, smoking, HTN, and depression. The more pronounced link observed in older individuals and those with specific lifestyles or experiencing depression indicates that LTPA should be prioritized for suicide prevention in these high-risk populations. The impact of age may be owing to a higher frequency of smoking, HTN, and depression in elderly individuals in our study. The more pronounced association between LTPA and suicidal ideation among individuals with lower BMI and sedentary time suggests slimming and avoidance of sedentary behaviors help to synergize the beneficial effect of LTPA. Smoking, HTN, and depression are potential risk factors for suicidal ideation, which are attributed to reduced serotonin levels or enhanced neuroinflammation [33, 34]. This may explain the enhanced effect of PA on suicidal ideation in these subgroups.

To our knowledge, we are the first to investigate the dose–response relationship between LTPA and suicidal ideation. According to our results, performing moderate intensity LTPA for 150–299 min/week is associated with lower possibility of suicidal ideation. Exceeding 300 min/week did not bring any additional benefits, and the safety for older people with chronic diseases or functional limitations is unclear. Two cohort studies investigating the associations between LTPA and depression have produced comparable results. In a cohort study conducted in a Korean population, with a median follow-up period of 3.72 years, engaging in 150–299 and > 300 min/week of LTPA was associated with a 38% and 44% reduction, respectively, in the risk of developing depression compared to those who did not engage in LTPA [35]. Another Brazilian cohort study comprising 12,709 adults indicated that any amount of LTPA was associated with a lower risk of incident depression over a four-year follow-up (1–149 min/week, RR: 0.65; 150–299 min/week, RR: 0.67; ≥300 min/week, RR: 0.61) [36]. Notably, our results do not imply that the threshold for suicidal ideation improvement is 150 min/week of moderate-intensity LTPA or its equivalent. Since the amount of PA was based on patients’ self-reported estimates rather than objective measures (e.g., physical activity monitors), we did not analyze PA as a continuous variable to determine precise thresholds.

Herein, neither OPA nor TPA was linked with suicidal ideation. LTPA, driven by intrinsic motivation, likely offers greater mental health benefits than OPA/TPA, which are often extrinsically motivated [37]. While Roh et al. found no TPA-suicidal ideation association in adolescents [38], prior studies suggest TPA may improve depressive symptoms [10, 11], warranting further validation.

There are several limitations in this study. First, the NHANES data was derived from a cross-sectional survey. Consequently, we were unable to investigate causal association between PA domains and suicidal ideation. To corroborate our findings, conducting other studies, such as prospective cohort and Mendelian randomization studies, is crucial to ascertain the possible impact of PA domains on suicidal ideation. Second, the PA domains evaluation was conducted using self-report questionnaires instead of objective measurements, and the amount of distinct PA domains was assessed for a typical week at a single time. We were unable to capture the stable levels or trajectories of PA domains. For instance, the median and IQR of TPA are both 0, which may not reflect small amounts of TPA. Further studies should collect consecutive data on PA domains by objective measurements to investigate the association of PA with suicidal ideation. Third, suicidal ideation was assessed by the item nine of the PHQ-9, which encompasses non-suicidal self-injury and may impact our outcomes. The accurate assessment of suicidal ideation by other tools is needed to verify the results. Fourth, confounding bias is unavoidable in observational studies. For instance, participants with suicidal ideation had higher ratio of smoking, hypertension, diabetes mellitus, hyperlipidemia, coronary heart disease, and stroke. Prior studies have demonstrated that lacking LTPA is associated with an increased risk of cardiometabolic diseases [39]. Thus, the adverse effect of lacking LTPA on suicidal ideation may related to those factors. Although the association between LTPA and suicidal ideation remained significant after adjusting for those factors in multivariable models, the interference of confounding factors should not be ignored.

## Conclusions

In this nationally representative cross-sectional survey of NHANES 2007–2018, we showed different relationships between domain-specific PA and suicidal ideation. LTPA, but not OPA or TPA, meeting the WHO PA guidelines was related to a lower risk of suicidal ideation and depression. The type (LTPA, OPA, and TPA), intensity (vigorous, moderate, or combination) and amount of PA should be assessed independently, and personalized recommendations must made. Collectively, we propose that increasing vigorous-intensity LTPA levels need to be advocated as a possible lifestyle change for suicidal ideation prevention or treatment. To validate our findings, conducting longitudinal studies as well as objective evaluations of PA is necessary.

## Supplementary Information

Supplementary Material 1.

## Data Availability

The datasets used in this study are publicly available in the National Health and Nutrition Examination Survey repository at https://wwwn.cdc.gov/nchs/nhanes/default.aspx. The datasets generated and analysed during the current study are available from the corresponding author on request.
