# Supplementary material for: Domain-specific physical activity and risk of suicidal ideation in adults: a population-based study
Source: BMC Public Health. 2025 Jul 29;25:2573. doi: 10.1186/s12889-025-23815-9 (PMC12305946; doi:10.1186/s12889-025-23815-9)
Supplement: Supplementary file 1 — Supplementary Material 1. [file 12889_2025_23815_MOESM1_ESM.docx]

**Domain-specific Physical Activity and Risk of Suicidal Ideation in Adults: A Population-based Study**

**Table S1** Logistic regression analysis to identify the association between domain-specific PA and depression

**Table S2** Ordinal logistic regression analysis to identify the association between domain-specific PA and the severity of suicidal ideation

**Figure S1** Multivariable OR for suicidal ideation based on the amount of PA. CI, confidence interval; OR, odds ratio; PA, physical activity. All ORs were adjusted for the same covariates in Model 2.

**Table S1** Logistic regression analysis to identify the association between domain-specific PA and depression

|  | **Unadjusted Model** | | **Model 1** | | **Model 2** | | |
| --- | --- | --- | --- | --- | --- | --- | --- |
|  | **OR (95% CI)** | ***P*-value** | **OR (95% CI)** | ***P*-value** | **OR (95% CI)** |  | ***P*-value** |
| **Total PA: achieved** | | | | | | | |
|  | 0.57 (0.51−0.64) | <0.001 | 0.65 (0.58−0.74) | <0.001 | 0.75 (0.66−0.86) |  | <0.001 |
| **Occupational PA: achieved** | | | | | | | |
|  | 0.82 (0.73−0.91) | <0.001 | 0.94 (0.84−1.05) | 0.30 | 0.92 (0.82−1.04) |  | 0.19 |
| **Transportation PA: achieved** | | | | | | | |
|  | 0.74 (0.64−0.86) | <0.001 | 0.86 (0.74−1.00) | 0.054 | 0.88 (0.75−1.04) |  | 0.12 |
| **Leisure-time PA: achieved** | | | | | | | |
|  | 0.50 (0.45−0.56) | <0.001 | 0.53 (0.47−0.59) | <0.001 | 0.64 (0.56−0.73) |  | <0.001 |

Model 1: adjusted for age, sex, and race.

Model 2: adjusted for age, sex, race, education level, marital status, family income-to-poverty ratio, body mass index, waist circumference, smoking, drinking, hypertension, diabetes mellitus, hyperlipidemia, coronary heart disease, stroke, cancer, and sedentary time.

CI, confidence interval; OR, odds ratio; PA, physical activity.

**Table S2** Ordinal logistic regression analysis to identify the association between domain-specific PA and the severity of suicidal ideation

|  | **N** | **PHQ 090 score** | | | **OR (95% CI)** |
| --- | --- | --- | --- | --- | --- |
|  |  | **1** | **2** | **3** |  |
| **PA: achieved** | 15,629 | 353 (2.0%) | 72 (0.4%) | 57 (0.3%) | 0.78 (0.63−0.97) |
| **OPA: achieved** | 8952 | 232 (2.4%) | 46 (0.4%) | 37 (0.4%) | 1.03 (0.84−1.28) |
| **TPA: achieved** | 3531 | 94 (2.2%) | 22 (0.5%) | 19 (0.5%) | 0.93 (0.69−1.26) |
| **LTPA: achieved** | 8641 | 141 (1.4%) | 29 (0.2%) | 24 (0.3%) | 0.69 (0.53−0.89) |

Adjusted for the same covariates in Model 2

LTPA, leisure-time physical activity; OPA, occupational physical activity; PA, physical activity; TPA, transportation physical activity.


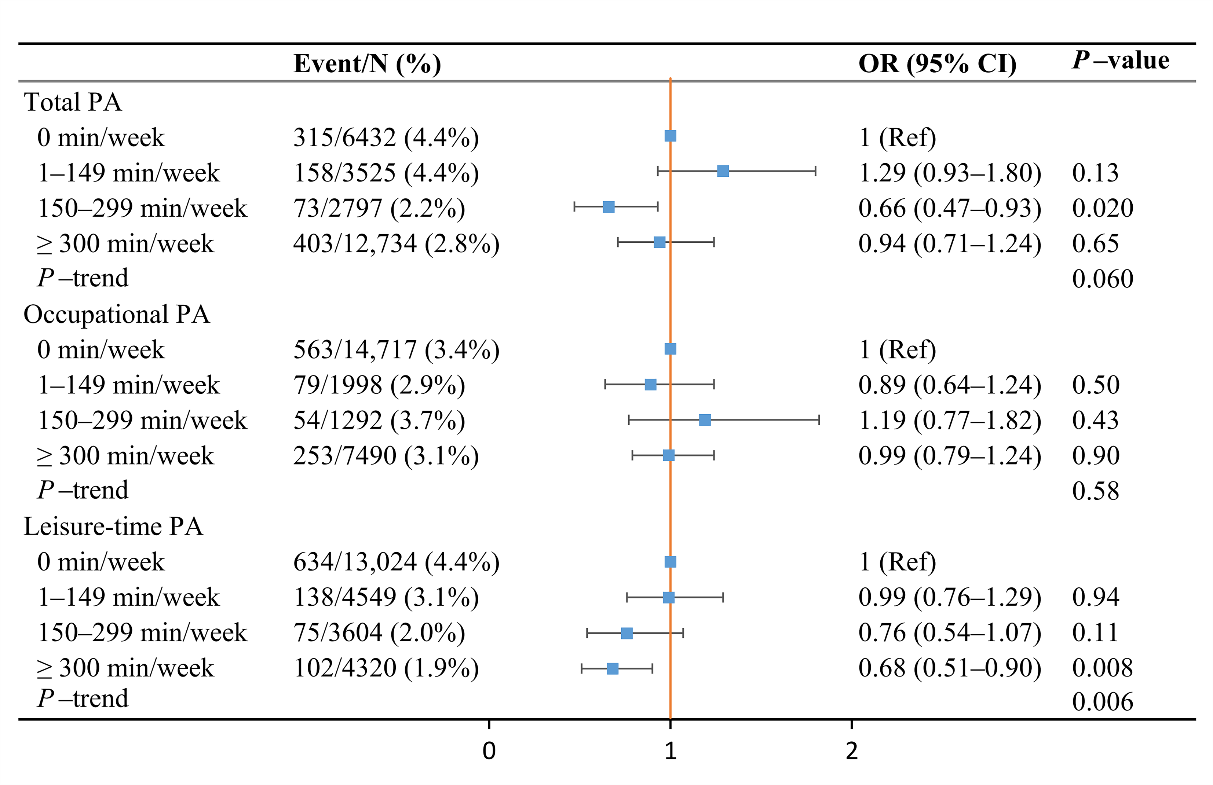


**Figure S1** Multivariable OR for suicidal ideation based on the amount of PA not applying the 2× weighting for vigorous-intensity PA. CI, confidence interval; OR, odds ratio; PA, physical activity. All ORs were adjusted for the same covariates in Model 2.
